# LogMPIE, pan-India profiling of the human gut microbiome using 16S rRNA sequencing

**DOI:** 10.1038/sdata.2018.232

**Published:** 2018-10-30

**Authors:** Ashok Kumar Dubey, Niyati Uppadhyaya, Pravin Nilawe, Neeraj Chauhan, Santosh Kumar, Urmila Anurag Gupta, Anirban Bhaduri

**Affiliations:** 1 Innovation Center, Tata Chemicals Ltd, Ambedveth, Pune, Maharashtra, 412111, India; 2 Thermo Fisher Scientific, Invitrogen BioServices India Pvt Ltd, Mumbai, Maharashtra, 400076, India; 3 Thermo Fisher Scientific, Life Science Solutions, Gurgaon, Haryana, 122016, India; 4 JSS Medical Research India Pvt. Ltd, Faridabad, Haryana, 121003, India; 5 JSS Medical Research India Pvt. Ltd, Mumbai, Maharashtra, 400086, India

**Keywords:** Metagenomics, Microbiome, DNA sequencing, Gastrointestinal system

## Abstract

The “Landscape Of Gut Microbiome - Pan-India Exploration”, or LogMPIE study, is the first large-scale, nationwide record of the Indian gut microbiome. The primary objective of the study was to identify and map the Indian gut microbiome baseline. This observational study was conducted across 14 geographical locations in India. Enrolled subjects were uniformly distributed across geographies (north, east, west and south) and body mass index (obese and non-obese). Furthermore, factors influencing the microbiome, such as age and physical activity, were also considered in the study design. The LogMPIE study recorded data from 1004 eligible subjects and reported 993 unique microorganisms across the Indian microbiome diaspora. The data not only map the Indian gut microbiome baseline but also function as a useful resource to study, analyse and identify signatures characterizing the physiological dispositions of the subjects. Furthermore, they provide insight into the unique features describing the Indian microbiome. The data are open and may be accessed from the European Nucleotide Archive (ENA) portal of the European Bioinformatics Institute (https://www.ebi.ac.uk/ena/data/view/PRJEB25642).

## Background & Summary

The gut microbiome and its host share a primarily symbiotic, commensal relationship that is occasionally pathogenic^1^. An increasing body of evidence now substantiates that the gut microbiome plays a critical role in digestion, nutrition, and immune system maturation^2–7^. A non-exhaustive list of physiological disorders associated with gut microbiome dysbiosis includes Crohn’s disease^8,9^, type II diabetes^10,11^, colorectal cancer^12,13^ and metabolic disorders^14,15^. With advancements in the gut microbiome field, modulation of host physiology and biochemistry by the microbiome is being investigated in greater detail^16^.

To better understand host physiology modulation by the gut microbiome, it is imperative to know the microbiome composition. Acknowledging this requirement, multiple consortia were set up to map the gut microbiome across different geographies. Pioneering efforts were initiated through the ‘The Human Microbiome Project’ (HMP)^17,18^ and Metagenome of Human Intestinal Tract (MetaHIT)^19,20^ studies. Following suit, multiple nationwide and cohort-specific studies were conducted to understand the impact of the gut microbiome. ^21,22^. These studies led to an exponential rise in available gut microbiome datasets across multiple cohorts.

Among the multiple factors contributing to the compositional diversity of the gut microbiome, two key influencers are geography and diet^23–28^. Comparative assessments across multiple populations, such as those between Europeans and Americans^19^, Koreans and other Asians^29^ and within Africans, confirmed that geography influences gut microbiome diversity^30,31^. Genome-scale metabolic simulations indicated that diet composition influences the growth rates of microorganisms within the gut^32^. The differential growth rate of these microorganisms leads to diversity within the gut microbiome^33,34^. Age as a factor was also reported to influence the gut microbiome composition^35,36^. An increase in gut microbiome diversity was most pronounced in infants^37^ and continued until adulthood^36,38^. Interestingly, the elderly population showed a loss in gut microbiome diversity^39^. Reduction in the gut microbiome diversity in the elderly population could be a result of dietary restrictions, constrained lifestyle, and medications^39^.

Over the past few years, multiple studies investigated sub-sections of the Indian gut microbiome^40–43^. Each of these gut microbiome studies did consider a cohort from different geographies and physiological dispositions. Unfortunately, owing to the lack of protocol consistency and processing pipeline variations, a meta-analysis based on the gut microbiome composite data was limited^44,45^. A comprehensive gut microbiome study describing the impact of geography, age, sex, BMI and physical activity across the Indian population has yet to be reported. A study along these lines would provide insight into the association of various factors, such as cultural affiliations, geography and changing lifestyle, with the gut microbiome composition.

The ‘Landscape Of Gut Microbiome - Pan-India Exploration’, or the LogMPIE, is to the best of our knowledge the first large-scale, observational, multi-centric, cross-geographic and diverse age group study focusing on the Indian population. This study reports data from 1004 participating subjects. The participants represented a uniform distribution of obese and non-obese subjects. Additionally, the study recorded sex and lifestyle patterns based on physical activity (sedentary and non-sedentary) of the participating subjects.

Adherence to a common standardized operating protocol and centralized sequencing facility reduced possibilities of sample processing and pipeline-related variations. Furthermore, cross-checking and validation at multiple points during sample and data processing assured strict quality control.

Based on a preliminary assessment, operational taxonomic unit (OTU) tables for the individual subjects were shared along with the FASTQ data. The sequence processing pipeline used thresholds for reporting higher specificity against a coverage trade-off (please refer to the Methods section for details). FASTQ data from the study are available from the European Nucleotide Archive portal of the European Bioinformatics Institute (Data Citation 1). Sharing of the FASTQ data enables users to re-compute OTU distributions using a preferred pipeline and customized parameters.

The LogMPIE study reported features specific to the Indian population. In comparison to the Western gut microbiome composition, the Indian gut microbiome reported a higher relative abundance of *Prevotella copri* (~0.39). Increased abundance of *Prevotella copri* is attributed to the high content of resistant starch within the standard Indian diet^46^. *Faecalibacterium prausnitzii* was the other copious microorganism reported with a relative abundance of ~0.13 (Table 1).

**Figure 1:**
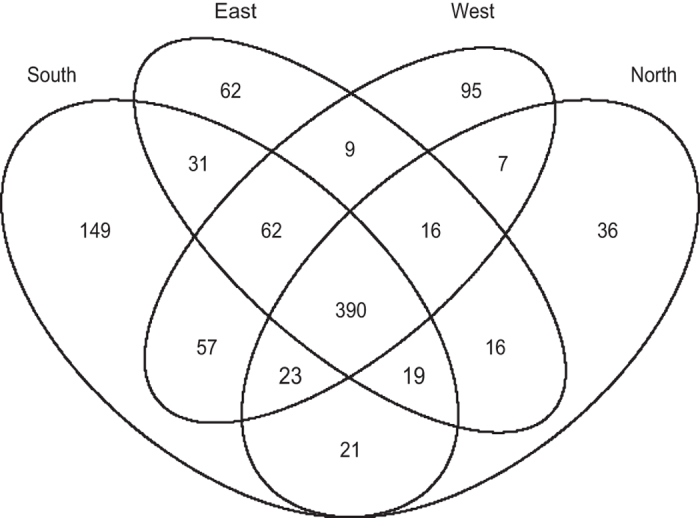
Distinct species reported across geographical locations. North (number of subjects 243), South (number of subjects 250), East (number of subjects 250) and West (number of subjects 261).

The comparative assessment indicated that 390 out of 993 microorganisms were present in all the geographical zones (Fig. 1). A detailed assessment of the gut microbiome composition and its variation owing to influencing factors is beyond the scope of the current article. However, the LogMPIE data allow an investigation of the gut microbiome composition in response to multiple influencing factors.

## Methods

The LogMPIE study was conducted across 14 geographical locations in India (Table 2). During the study, all pertinent requirements recommended by the Indian Council of Medical Research (ICMR) for Biomedical Research on Human Subjects and by the International Conference on Harmonization-Good Clinical Practice (ICH-GCP) were consulted and adhered to. The study was registered with the Clinical Trial Registry-India (CTRI Number: CTRI/2016/03/007616). Following acceptance of the study protocol by independent ethics committees/institutional review boards (IEC/IRB), the LogMPIE study was initiated. Prior to initiation of the study, willing volunteers from the 14 geographical locations were educated on the study objectives and the sampling procedure, and consent documents were obtained.

### Study Design

**Figure 2:**
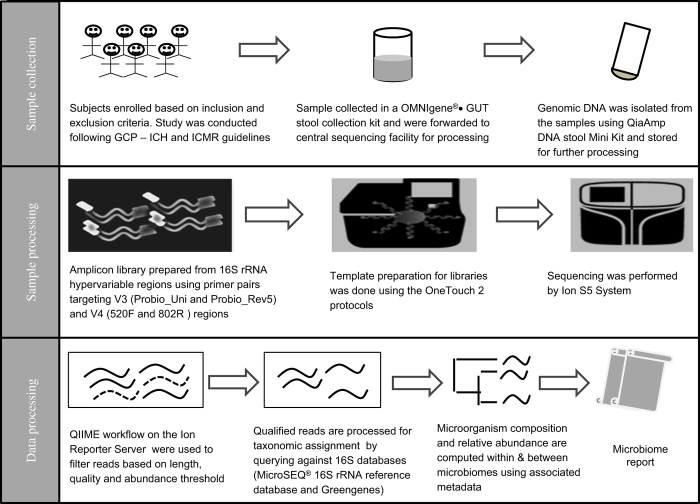
Schematic workflow elucidating sample collection, sample processing and data processing adopted during the LogMPIE study.

Since LogMPIE was an observational, multi-centric and non-interventional study, the sample size of the study was not statistically derived. Enrolment of subjects for the study was based on the inclusion and exclusion criteria, as listed in Table 3. A schema of the study workflow is included in Fig. 2.

A total of 1022 subjects were enrolled and screened. The data from 1004 eligible subjects were obtained, processed and reported. Subject distributions were guided based on geography and body mass index (with obese and non-obese being the two groups) uniformity. Details of subject distribution under individual categories are reported in Table 4. Subject classification under the physical activity category (sedentary and non-sedentary) was based on features adapted from the MOPO study^47^. Furthermore, the grouping of subjects under the BMI category was based on the recommendations from the National Heart, Lung and Blood Institute^48^.

### Sample Collection

Faecal samples were collected in a pre-labelled sterile OMNIgene®•GUT stool collection kit (OMR-200, DNA Genotek, Ottawa, Canada) by individual subjects^49^. The stool collection kit is an all-in-one system (for details please refer to Supplementary Information, S1.1). It is designed to stabilize and maintain DNA integrity, thus enabling gut microbiome profiling at an ambient temperature.

Prior to stool collection, adequate training and instructions regarding the collection process were provided to individual subjects. Samples collected in the OMNIgene®•GUT stool collection kit were stored at ~20 °C. This temperature adhered to the kit manufacturer’s instructions. The temperature was maintained at ~20°C until sample processing at the central sequencing facility. The samples were processed for genomic DNA isolation within 2 days of collection.

### DNA Isolation

The bacterial genomic DNA was isolated and purified from the collected faecal samples using a QiaAmp DNA Stool Mini Kit (Qiagen, Hilden, Germany). ASL buffer was used to lyse the stool samples. Normally, bacterial cells lyse at 70°C in the ASL buffer. For the current protocol, a higher lysis temperature (95°C) was used to account for cells that were difficult to lyse (such as Gram-positive bacteria). Post lysis, DNA-damaging agents and PCR inhibitors were removed using InhibitEX Matrix Tablets (Qiagen, Hilden, Germany). A standardized protocol was used to isolate DNA using QIAamp Mini Spin Columns (Supplementary Information, S1.2). The genomic DNA was eluted in 100 μl of elution buffer. The quality of the isolated genomic DNA was confirmed by agarose gel electrophoresis. DNA concentration was estimated with a Qubit 2.0 instrument and with a Qubit dsDNA HS Assay Kit (Thermo Fisher Scientific, Carlsbad, CA, USA). A detailed standardized protocol adopted for DNA quantification is included within the supplementary section (Supplementary Information, S1.3).

### 16S Primers and Amplicon Library Generation

The hypervariable regions of the 16S rRNA gene were PCR amplified using extracted DNA as the template. For details regarding the protocol, please refer to the supplementary section (Supplementary Information, S1.4). Primer pair Probio_Uni and Probio_Rev were used to amplify the V3 region^50^. To target the V4 region of the 16S rRNA gene, a primer pair of 520F and 802R was used^51^. Primer details are listed in Table 5.

Amplitaq Gold 360 MM (Thermo Fisher Scientific, Foster City, CA, USA) was used for 16S rRNA gene amplification, and the PCR conditions were set as follows: initial denaturation at 94°C for 5 min, denaturation at 94°C for 30 sec, annealing at 55°C for 30 sec and extension at 72°C for 90 sec. The final extension was performed at 72°C for 10 min. PCR amplification was performed using a 9700 Thermocycler (Thermo Fisher Scientific, Grand Island, NY, USA). The PCR amplicon was purified using AMPure XP reagent (Beckman Coulter, Brea, CA, USA). The concentration of the amplicon was determined with a Qubit dsDNA HS Assay Kit. The respective size distribution of the amplicon was verified with an Agilent 2100 Bioanalyzer using a high-sensitivity DNA kit (Agilent, Santa Clara, CA, USA). The amplicon library was diluted to 100 pM, and an equimolar pool was prepared for clonal amplification. Protocols used for amplicon library formation are included in the supplementary section (Supplementary Information, S1.3).

### Template Preparation and Sequencing

Template preparation for libraries using ion spheres was performed using OneTouch 2 protocols and corresponding reagents (Thermo Fisher Scientific, Carlsbad, CA, USA). The Ion OneTouch 2 system (Thermo Fisher Scientific, Carlsbad, CA, USA) enables automated delivery of Ion Sphere Particle (ISP) templates. Further details regarding the Ion One Touch 2 system are included within the supplementary section (Supplementary Information, S1.5). The ISP templates were loaded either on an Ion 520 or Ion 530 chip kit, and a standardized protocol was followed. Sequencing was performed with the Ion 520 or Ion 530 kit-OT2 (Thermo Fisher Scientific, Carlsbad, CA, USA) using the 200 bp chemistry with 500 flow (125 cycles) for the V3 region and 400 bp chemistry with 800 flow (200 cycles) for the V4 region run format. The sequencing was performed on an Ion S5 System. Using the default pre-processing parameters, reads pertaining to adaptor sequences were filtered out, and the sequence data were stored in the FASTQ format. Individual sequence data for the subjects are available at the European Nucleotide Archive (Data Citation 1).

### Bioinformatics Analysis

The raw sequencing data were processed through a customized 16S analysis pipeline to report the taxonomical distribution of species along with their relative abundance in an OTU table (Relative Abundance Table, Data Citation 2). The customized processing was performed using a QIIME workflow on the Ion Reporter Server^51^. It should be noted that the content of the OTU table depends on the pipeline and the parameters used for processing the data. For customized assessments, users are encouraged to regenerate the OTU table from the shared FASTQ data, employing their preferred pipeline and parameters.

### Pre-processing Sequence Data

The sequence data were pre-processed, and the step primers were trimmed. The minimum read length threshold was set to 100 bp. Reads recording lengths shorter than the threshold were dropped from further processing. Read sequences were clustered together and checked for copy number with a minimum threshold of 10 reads. Low copy numbers (threshold <10) were filtered out and dropped from further analysis.

### Organism Screening and Assessment

The reads were aligned against two comprehensive 16S databases, the Thermo Fisher Scientific in-house MicroSEQ® 16S rRNA reference database (V2013.1) and the curated Greengenes database (V13.5)^52^. Reads were aligned against the databases using Megablast (from the BLAST package). The expectation value (E-value) for the searches was set to 0.01, and the max target hits value was set to 100^53^. To assign taxonomy, the minimum alignment percentage of a read to a subject sequence (homologue) in the database was set to a threshold of 90. A read was assigned to a genus only when the identity score of the sequence alignment (between the read and subject sequence from the database) was at 97% or higher. For species assignment, the minimum percentage identity of the alignment was set to 99%.

Reads assigned to multiple entities (genus or species) at a taxonomy level were further assessed for refinement. In a scenario where the top homologue of a read (the most similar sequence based on the Megablast search) reports a sequence identity of greater than 0.2% in comparison to the next homologue, the read was assigned the taxonomic label of the top homologue. On failure, the read was assigned the taxonomic label of the homologues within 0.2% of the top homologue. For reads with a conflicting assignment, a “slash ID” was issued. The slash ID recorded the multiple taxonomy assessments.

The taxonomy distribution counts or abundance was derived from the clustered reads. The abundance value from the pipeline was further transformed into the relative abundance of the individual species. The shared OTU table (Relative Abundance Table, Data Citation 2) was derived using a QIIME workflow on the Ion Reporter Server and in-house optimized parameters.

### Data Analysis Pipeline

Customized assessment of the LogMPIE data was performed using Ion Reporter^TM^ software (please refer to the previous sub-sections for the parameters used in the analysis). To use Ion Reporter^TM^ software, individual users are required to register with the portal (https://ionreporter.thermofisher.com/ir/). For standalone processing of the data, ‘Microbiome Processing Pipeline’, a Python-based tool, is being shared at GitHub (https://github.com/anirbanbhaduri/LogMPIE). Based on user-defined parameters, the pipeline processes input FASTQ data and reports out OTU tables. The tool enables a user to download the microbiome processing tools (QIIME^54^ and Mothur^55^). For taxonomic referencing, the tool may use Greengenes^56^, Silva^57^, and RDP^58^ databases. Owing to licensing implications, processing tools and databases need to be obtained separately by the user.

## Data Records

The LogMPIE study repository shares 3 data types (explained below). The data are organized to enable multiple forms of assessments. Data type 1 is available at the European Nucleotide Archive (ENA) portal of European Bioinformatics Institute, while the other 2 data types are shared through the Supplementary Information.

### Data type 1

FASTQ data obtained from sequencing the V3 and V4 regions of the 16S rRNA gene of microorganisms hosted within individual subjects are shared as a part of the LogMPIE study repository. The data comprises 1004 FASTQ sequence files (Data Citation 1). They are found under the primary accession code, PRJEB25642, and secondary accession code, ERP07577, on the ENA portal. These FASTQ files were processed through a QIIME workflow on the Ion Reporter Server^51^. It should be noted that FASTQ files enable users to customize their assessments based on selected parameters and pipelines.

### Data type 2

The OTU table reporting the relative abundance of microorganism across individuals is available as Supplementary Information (Relative Abundance Table, Data Citation 2). The table reports the relative abundance of all microorganisms at the species level. The strain information is currently not included but may be obtained through a customized FASTQ data processing pipeline by interested users.

### Data type 3

Data type 3 reports the study metadata (LogMPIE Study Metadata, Data Citation 2). This comprises the codes of participating subjects along with information regarding their age, sex, physical activity, BMI and geographical locations. Attributes within the metadata would facilitate retrospective studies.

## Technical Validation

Several layers of quality assurance and quality control (QC) systems were implemented and maintained. To ensure that the study was conducted and the data were generated, documented and reported in compliance with the ICH-GCP and ICMR guidelines for Biomedical Research on Humans, standard operating protocols were developed. Furthermore, each individual working on the study was trained on the protocols.

### Sample Management

Iterative data checks were performed to ensure accuracy in data entry. Manual inspection of the data integrity within the database was performed by the QC team before the database was locked.

### DNA Sample Quality Control

All the isolated DNA samples were quantified, checked for quality and further used for the amplicon library preparation. 16S rRNA gene amplicon generation success was assessed by reviewing the amplicon size. The primer pair Probio_Uni and Probio_Rev (V3 Region) led to a PCR product of 194 bp, and the primer pair 520 F and 802 R (V4 region) produced a PCR product of 263 bp according to the *Escherichia coli* K-12 16S rRNA gene sequence. The absence of contaminants and the respective size distribution of the amplicon were verified using an Agilent 2100 Bioanalyzer and the Qubit dsDNA HS Assay Kit.

### Sequence Quality Assessment and Bioinformatics Pipeline

**Figure 3:**
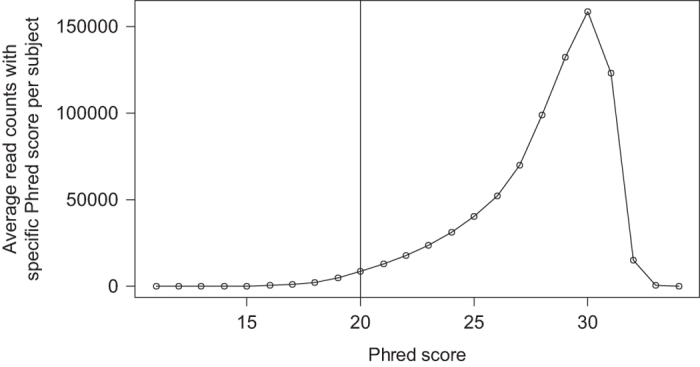
Plot of the Phred score against the ‘Average read counts with specific Phred score per subject’, across the 1004 subjects. Phred score threshold for the taxonomic assignment was set to 20.

Reads were further assessed based on the quality score. Histograms of the Phred scores for all the reads of the 1004 FASTQ samples are shown in Fig. 3. Geographical location wise plots are shared as Supplementary Information (Supplementary Information, Figure S1). The FASTQ data may be refined based on the Phred score. In the current taxonomic assignment, a Phred score threshold of 20 was considered to reduce both noise and false positives in the data. Using a minimum read length of 100 bp and having a threshold of 10 reads per cluster, reliable read data were obtained. These data were processed and used to generate the OTU table. Parameters used to process the data are sensitive and would influence the produced OTU table.

## Usage Notes

The primary objective of the LogMPIE study was to report the Indian gut microbiome composition baseline. Additionally, the study recorded geographical location, sex, age, physical activity and body mass index for each participating subject. The repository, to the best of our knowledge, is the most comprehensive gut microbiome dataset representing the Indian population. The authors acknowledge that this study, though comprehensive, may not be exhaustive.

The LogMPIE study acts as a powerful microbiome dataset to enable multiple applications. In addition, the dataset helps to identify and quantify features or descriptors associated with physiological dispositions of the host.

## Additional information

**How to cite this article:** Dubey, A. K. *et al.* LogMPIE, pan-India profiling of the human gut microbiome using 16S rRNA sequencing. *Sci. Data*. 5:180232 doi: 10.1038/sdata.2018.232 (2018).

**Publisher’s note:** Springer Nature remains neutral with regard to jurisdictional claims in published maps and institutional affiliations.

## Supplementary Material



Supplementary Information

## Figures and Tables

**Table 1:** Relative abundance and frequency of observation in the study sample of the top 10 microorganisms within the study cohort.

Organisms (order; family)	Relative Abundance	Frequency of Observation in the Study Sample
*Prevotella copri* (o = Bacteroidales; f = Prevotellaceae)	0.391	0.966
*Faecalibacterium prausnitzii* (o = Clostridiales; f = Ruminococcaceae)	0.131	0.966
*Bacteroides plebeius* (o = Bacteroidales; f = Bacteroidaceae)	0.041	0.964
*Haemophilus parainfluenzae* (o = Pasteurellales; f = Pasteurellaceae)	0.033	0.861
*Roseburia faecis* (o = Clostridiales; f = Lachnospiraceae)	0.025	0.962
*Megasphaera elsdenii* (o = Veillonellales; f = Veillonellaceae)	0.024	0.933
*Lactobacillus rogosae* (o = Lactobacillales; f = Lactobacillaceae)	0.022	0.964
*Prevotella stercoreacopri* (o = Bacteroidales; f = Prevotellaceae)	0.021	0.964
*Parasutterella excrementihominis* (o = Burkholderiales; f = Sutterellaceae)	0.020	0.924
*Ruminococcus gnavus* (o = Clostridiales; f = Lachnospiraceae)	0.017	0.958

**Table 2:** Sample counts from the multiple study centres.

Site	Geographical Location	Number of Samples
Bhopal	North	65
Ludhiana	North	65
Lucknow	North	67
New Delhi	North	46
Guwahati	East	83
Kolkata	East	84
Patna	East	83
Ahmedabad	West	65
Ajmer	West	70
Mumbai	West	59
Nagpur	West	67
Chennai	South	89
Cochin	South	96
Mangalore	South	65

**Table 3:** Inclusion and exclusion criteria of the study.

Inclusion Criteria	Exclusion Criteria
• Age: 18-65• Certified healthy on physical examination and free from diabetes, acquired immunodeficiency syndrome (AIDS), chronic diarrhoea, inflammatory bowel disease, irritable bowel syndrome, or other gastrointestinal disorders, gastrointestinal surgery [with exception of appendectomy, polypectomy, or herniorrhaphy].	• Prescription, OTC medications or supplements (e.g., acid anti-secretory drugs, probiotics) known to alter the gut function or microbiome during the 4 weeks prior to study enrolment

**Table 4:** Distribution of subjects across different study categories.

Categories	Distribution
Age	Range (Years): **18–65**
Sex	Male = **591**; Female = **431**
BMI	Underweight = **31** Normal = **263** Overweight = **277** Obese = **451**
Geographical Location	North = **247** South = **262** East = **250** West = **263**
Physical Activity	Sedentary = **470** Non-sedentary = **552**

**Table 5:** Details of the primers used to amplify the 16S rRNA gene.

Primer Name	Adapter Sequence	Key	Barcode	Barcode Adapter	Primer Sequence (5′-3′)
Probio_Uni^50^	CCATCTCATCCCTGCGTGTCTCCGAC	TCAG	TTACAACCTC	GAT	CCTACGGGRSGCAGCAG
Probio_Rev^50^	CCTCTCTATGGGCAGTCGGTGAT				ATTACCGCGGCTGCT
520F^51^	CCATCTCATCCCTGCGTGTCTCCGAC	TCAG	TTACAACCTC	GAT	AYTGGGYDTAAAGNG
802R^51^	CCTCTCTATGGGCAGTCGGTGAT				TACNVGGGTATCTAATCC

## References

[d1] European Nucleotide Archive 2018 PRJEB25642

[d2] figshare Dubey A. K. *et al.* 2018 https://doi.org/10.6084/m9.figshare.c.4147079

