# Supplementary material for: LogMPIE, pan-India profiling of the human gut microbiome using 16S rRNA sequencing
Source: Sci Data. 2018 Oct 30;5:180232. doi: 10.1038/sdata.2018.232 (PMC6207063; doi:10.1038/sdata.2018.232)
Supplement: Supplementary Information [file sdata2018232-s2.docx]

Title

LogMPIE, pan-India exploration of the human gut microbiome using 16S rRNA sequencing

File type

Supplementary Materials

Contents

[S1. Sample collection and processing protocols 2](#_Toc517863854)

[S1.1 Stool collection kit 2](#_Toc517863855)

[S1.2 DNA isolation protocol 2](#_Toc517863856)

[S1.3 DNA Quantification protocol 2](#_Toc517863857)

[S1.4 The 16S Primers design and Amplicon Library Generation 2](#_Toc517863858)

[S1.5 Ion One Touch 2 protocol 2](#_Toc517863859)

[S2. Glossary of tools, kits and instruments 4](#_Toc517863860)

[S3 Geography based Phred score distribution plot 5](#_Toc517863861)

# S1. Sample collection and processing protocols

Sample collection and processing were performed in accordance to standardized protocols as advised by the manufacturer. Links to the detailed protocols are being provided below. Any deviations from the standardized protocol were already discussed as a part of the main text.

## S1.1 Stool collection kit

OMNIgene®•GUT stool collection kit (OMR-200, DNA Genotek, Ottawa, Canada) was used to collect stool samples. The stool collection kit is an all-in-one system. It is designed to stabilize, retain DNA integrity and profile microbiome of fecal samples at ambient temperature during transportation. Details of the collection process are discussed within the kit’s data sheet (<https://www.dnagenotek.com/us/pdf/PD-BR-00159.pdf>).

## S1.2 DNA isolation protocol

Bacterial genomic DNA was isolated and purified from the collected fecal sample using the QiaAmp DNA Stool Mini Kit. Details of the protocol may be obtained from the QIAamp® DNA Stool Handbook (<https://www.qiagen.com/us/resources/download.aspx?id=e5f66d36-1f1c-4e89-828e-257088bdad53&lang=en>)

## S1.3 DNA Quantification protocol

Quantification of the isolated DNA was performed using the Qubit dsDNA HS Assay kit and the Qubit 2.0 instrument. Details of the protocol may be obtained from the “Qubit® dsDNA HS Assay Kit” user guide (<https://assets.thermofisher.com/TFS-Assets/LSG/manuals/Qubit_dsDNA_HS_Assay_UG.pdf>)

## S1.4 The 16S Primers design and Amplicon Library Generation

A fusion PCR approach was used to prepare an amplicon library. The approach requires two fusion primers (forward and reverse primer) per target region. The hypervariable regions of the 16S rRNA gene were amplified from the extracted DNA. Details regarding fusion primers design are available at

<https://dna.uga.edu/wp-content/uploads/sites/51/2013/12/Incorporating-Barcodes-into-Amplicon-Fusion-Primer-Design.pdf>.

The PCR amplicon was purified using AGENCOURT® AMPURE® (Beckman Coulter) protocol. Details of the protocol may be obtained at: <https://genome.med.harvard.edu/documents/sequencing/Agencourt_AMPure_Protocol.pdf>

## S1.5 Ion One Touch 2 protocol

Ion One Touch 2 System simplifies the workflows for the Ion S5 system to provide an automated solution for scalable and reproducible template preparation. The Ion OneTouch 2 system works on the three breakthrough technologies that enable automated delivery of template Ion Sphere Particles. The first is the reaction filter that creates millions of microreactors in which clonal amplification occurs. The second is the fully integrated thermal cycler and disposable path amplification plate system that enables robust thermal cycling of the microreactors. The third is the integrated centrifuge, which recovers the template Ion Sphere particles. The ion sphere particle (ISP) templates were loaded on either Ion 520 or Ion 530 chip kit.

Details regarding the template preparation on Ion One Touch may be obtained at Ion 520™ & Ion 530™ Kit – OT2 user guide (<https://assets.thermofisher.com/TFS-Assets/LSG/manuals/MAN0010844_Ion_520_530_OT2_UG.pdf>)

Details related to OneTouch 2 protocols are included Ion OneTouch™ 2 System user guide (<https://assets.thermofisher.com/TFS-Assets/LSG/manuals/MAN0014388_IonOneTouch2Sys_UG.pdf>).

# S2. Glossary of tools, kits and instruments

**OMNIgene GUT stool collection kit:** Self-collection kits to collect and stabilize DNA for gut microbiome profile analysis

**QiAmp DNA stool Mini Kit:** Qiagen DNA Stool Mini Kit enable DNA purification stool samples. The sample could be frozen or fresh.

**Qubit dsDNA HS Assay kit:** Thermo Fisher Scientific kit designed specifically for use with the Qubit Fluorometer. The kit provides concentrated assay reagent, dilution buffer, and pre-diluted DNA standards.

**Amplitaq Gold 360 MM:** AmpliTaq Gold 360 PCR Master Mix is a Thermo Fisher Scientific kit containing solution enabling PCR amplification.

**Agencourt AMPure XP:** Automated PCR purification system enabling DNA extraction from Beckman Coulte .

**One Touch 2 protocols:** Thermo Fisher Scientific protocol performing template amplification and enrichment as a part of the manual workflow for the Ion PGM, Ion Proton, and Ion S5 and Ion S5 XL systems. It provides scalable template preparation for all Ion semiconductor chips.

**Ion 520 and Ion 530 kit-OT2:** Thermo Fisher Scientific kit enabling accurate and reproducible template preparation and sequencing of up to 400 base-read libraries using the Ion OneTouch 2 and the Ion S5 or Ion S5 XL Sequencing Systems.

**Ion Reporter Sequencing Software:** Ion Reporter Software provides an optimized suite of simple data analysis tools that streamline Ion GeneStudio S5 Systems, Ion PGM, and Ion Proton systems data analysis, so you can focus on finding the biological meaning of your data, and less on configuring and setting up software.

**HS Chip assay:** A sensitive ChIP kit designed to allow high quality DNA enrichment from as little as 1,000 cells per ChIP reaction for abundant targets.

**Ion PGM Hi-Q View OT2 Kit:** The Ion PGM Hi-Q View OT2 Kit enables accurate and reproducible template preparation for libraries up to 400 base pairs using the Ion OneTouch 2 System and is compatible with the Ion PGM System.

**Ion PGM Hi-Q View Sequencing Kit**: The Ion PGM Hi-Q View Sequencing Kit contains reagents and consumables for robust and highly accurate sequencing of 200 and 400 base pair libraries using the Ion OneTouch 2 System combined with the Ion PGM System.

# S3 Geography based Phred score distribution plot

**Figure S1:** Phred score distribution plot, as observed in the 16S sequenced FASTQ files. The FASTQ files were obtained by sequencing the isolated 16S DNA from the stool samples of 1004 subjects. Samples form the subjects were obtained from 14 geographical locations across India. In the assessment minimum quality score threshold was set to Phred 20.
